# Supplementary material for: Association of Polymorphisms in the Long Non-Coding RNA HOTAIR with Recurrent Pregnancy Loss in a Korean Population
Source: Genes (Basel). 2022 Nov 17;13(11):2138. doi: 10.3390/genes13112138 (PMC9690393; doi:10.3390/genes13112138)
Supplement: Supplementary file 1 [file genes-13-02138-s001.zip › genes-1985469-supplementary.pdf]

Table S1. Comparison of genotype frequencies and AOR values of polymorphism between the RPL and control subjects.

Genotypes	Controls (n=383)	PL = 2 (n=192)	AOR (95% CI)	*P*	FDR	PL≥3 (n=211)	AOR (95% CI)	P^a	P^b	PL≥4 (n=84)	AOR (95% CI)	P^a	P^b
HOTAIR **rs4759314**													
AA	351 (91.6)	155 (80.7)	1.000 (reference)			168 (79.6)	1.000 (reference)			67 (79.8)	1.000 (reference)		
AG	30 (7.8)	36 (18.8)	2.741 (1.628-4.614)	0.0001	0.0004	43 (20.4)	3.054 (1.847-5.050)	<0.0001	<0.0001	17 (20.2)	3.236 (1.675-6.251)	0.0005	0.002
GG	2 (0.5)	1 (0.5)	1.129 (0.102-12.549)	0.921	0.921	1 (0.5)	1.095 (0.098-12.175)	0.941	0.937	0 (0.0)	N/A	N/A	N/A
Dominant (AA vs AG+GG)			2.640 (1.585-4.397)	0.0002	0.0008		2.933 (1.792-4.801)	<0.0001	<0.0001		3.020 (1.575-5.793)	0.0009	0.004
Recessive (AA+AG vs GG)			0.996 (0.090-11.053)	0.997	0.997		0.947 (0.085-10.522)	0.965	0.965		N/A	N/A	N/A
HOTAIR **rs920778**													
TT	236 (61.6)	105 (54.7)	1.000 (reference)			121 (57.3)	1.000 (reference)			50 (59.5)	1.000 (reference)		
TC	134 (35.0)	71 (37)	1.218 (0.842-1.764)	0.296	0.536	78 (37.0)	1.141 (0.799-1.627)	0.468	0.756	29 (34.5)	1.013 (0.611-1.678)	0.961	0.961
CC	13 (3.4)	16 (8.3)	2.794 (1.297-6.018)	0.009	0.035	12 (5.7)	1.897 (0.830-4.335)	0.129	0.516	5 (6.0)	1.889 (0.641-5.567)	0.249	0.614
Dominant (TT vs TC+CC)			1.354 (0.952-1.925)	0.092	0.184		1.203 (0.854-1.694)	0.291	0.558		1.085 (0.670-1.758)	0.741	0.741
Recessive (TT+TC vs CC)			2.604 (1.225-5.539)	0.013	0.052		1.750 (0.782-3.918)	0.174	0.592		1.861 (0.643-5.391)	0.252	0.734
HOTAIR **rs1899663**													
GG	224 (58.5)	107 (55.7)	1.000 (reference)			128 (60.7)	1.000 (reference)			57 (67.9)	1.000 (reference)		
GT	139 (36.3)	72 (37.5)	1.338 (0.650-2.753)	0.430	0.536	75 (35.5)	0.921 (0.645-1.316)	0.651	0.756	22 (26.2)	0.612 (0.357-1.049)	0.074	0.148
TT	20 (5.2)	13 (6.8)	1.118 (0.773-1.616)	0.554	0.921	9 (4.3)	0.756 (0.331-1.726)	0.506	0.675	5 (6.0)	0.920 (0.328-2.581)	0.874	0.995
Dominant (GG vs GT+TT)			1.380 (0.661-2.883)	0.391	0.522		0.902 (0.640-1.273)	0.558	0.558		0.648 (0.310-1.073)	0.092	0.184
Recessive (GG+GT vs TT)			1.150 (0.809-1.636)	0.436	0.871		0.800 (0.357-1.794)	0.589	0.785		1.124 (0.408-3.093)	0.821	0.995
HOTAIR **rs7958904**													
GG	210 (54.8)	110 (57.3)	1.000 (reference)			122 (57.8)	1.000 (reference)			51 (60.7)	1.000 (reference)		
GC	144 (37.6)	66 (34.4)	0.889 (0.612-1.290)	0.536	0.536	78 (37.0)	0.945 (0.662-1.349)	0.756	0.756	29 (34.5)	0.825 (0.498-1.364)	0.453	0.604
CC	29 (7.6)	16 (8.3)	1.069 (0.556-2.054)	0.842	0.921	11 (5.2)	0.678 (0.326-1.413)	0.300	0.300	4 (4.8)	0.566 (0.190-1.685)	0.307	0.614
Dominant (GG vs GC+CC)			0.919 (0.647-1.305)	0.637	0.637		0.896 (0.638-1.260)	0.529	0.558		0.782 (0.483-1.267)	0.317	0.423
Recessive (GG+GC vs CC)			1.122 (0.593-2.122)	0.724	0.965		0.682 (0.333-1.397)	0.296	0.592		0.610 (0.209-1.786)	0.367	0.734

[a] Fisher's exact test.

[b] False discovery rate-adjusted *P* value for multiple hypotheses testing using the Benjamini-Hochberg method. Acceptance of statistical significance at $P< 0.05$ and 95 % CI not including 1.

Note: For AOR was adjusted by age of participants. RPL=recurrent pregnancy loss;
AOR=adjusted odds ratio; CI=confidence interval.

Table S2. Haplotype analysis of *HOTAIR* polymorphisms in RPL and controls subjects.

Haplotype	Controls (2n=766)	Case (2n=806)	OR (95% CI)	*P*[*]
rs4759314A>G/rs920778 T>C/rs1899663 G>T/rs7958904 G>C				
A-T-G-G	0.689	0.577	1.000 (reference)	
A-T-G-C	0.007	0.039	7.040 (2.715-18.260)	<0.0001
A-T-T-G	0.005	0.025	5.677 (1.926-16.730)	0.001
A-T-T-C	0.070	0.044	0.757 (0.487-1.175)	0.214
A-C-G-G	0.025	0.071	3.406 (1.997-5.811)	<0.0001
A-C-G-C	0.002	0.010	4.542 (0.959-21.500)	0.053
A-C-T-G	0.006	0.015	3.406 (1.091-10.640)	0.040
A-C-T-C	0.151	0.116	0.920 (0.682-1.241)	0.586
G-T-G-G	0.009	0.036	4.704 (2.041-10.840)	<0.0001
G-T-G-C	0.009	0.007	0.973 (0.324-2.917)	0.961
G-T-T-G	0.000	0.005	10.220 (0.548-190.400)	0.049
G-T-T-C	0.001	0.012	11.350 (1.447-89.080)	0.004
G-C-G-G	0.002	0.008	3.974 (0.821-19.230)	0.092
G-C-G-C	0.022	0.016	0.868 (0.417-1.807)	0.853
G-C-T-G	0.000	0.016	30.650 (1.816-517.400)	<0.0001
G-C-T-C	0.000	0.001	3.406 (0.138-83.880)	0.469
rs4759314A>G/rs920778 T>C/rs1899663 G>T				
A-T-G	0.699	0.614	1.000 (reference)	
A-T-T	0.072	0.071	1.124 (0.761-1.661)	0.555
A-C-G	0.028	0.079	3.255 (1.957-5.415)	<0.0001
A-C-T	0.157	0.134	0.977 (0.732-1.302)	0.871
G-T-G	0.015	0.044	3.551 (1.788-7.054)	<0.0001
G-T-T	0.005	0.017	3.798 (1.241-11.620)	0.016
G-C-G	0.024	0.028	1.256 (0.671-2.350)	0.474
G-C-T	0.000	0.013	24.950 (1.466-424.900)	<0.0001
rs4759314A>G/rs920778 T>C/rs7958904 G>C				

A-T-G	0.683	0.754	1.000 (reference)	
A-T-C	0.077	0.013	0.161 (0.083-0.309)	<0.0001
A-C-G	0.036	0.030	0.739 (0.422-1.290)	0.285
A-C-C	0.163	0.152	0.841 (0.638-1.108)	0.217
G-T-G	0.008	0.004	0.431 (0.107-1.732)	0.317
G-T-C	0.011	0.000	0.051 (0.002-0.881)	0.002
G-C-G	0.002	0.006	4.308 (0.501-37.010)	0.226
G-C-C	0.020	0.041	1.777 (0.967-3.266)	0.061
rs4759314A>G/rs1899663 G>T/rs7958904 G>C				
A-G-G	0.714	0.648	1.000 (reference)	
A-G-C	0.009	0.048	5.827 (2.583-13.150)	<0.0001
A-T-G	0.011	0.041	4.314 (1.974-9.429)	<0.0001
A-T-C	0.221	0.159	0.788 (0.608-1.020)	0.070
G-G-G	0.011	0.043	4.067 (1.936-8.546)	<0.0001
G-G-C	0.031	0.025	0.872 (0.476-1.597)	0.656
G-T-G	0.000	0.021	36.600 (2.194-610.700)	<0.0001
G-T-C	0.002	0.014	11.500 (1.479-89.470)	0.003
rs920778 T>C/rs1899663 G>T/rs7958904 G>C				
T-G-G	0.697	0.613	1.000 (reference)	
T-G-C	0.016	0.046	3.077 (1.616-5.857)	<0.0001
T-T-G	0.006	0.031	6.756 (2.334-19.560)	<0.0001
T-T-C	0.072	0.056	0.884 (0.585-1.336)	0.559
C-G-G	0.028	0.079	3.145 (1.908-5.183)	<0.0001
C-G-C	0.024	0.026	1.195 (0.634-2.249)	0.581
C-T-G	0.005	0.032	6.756 (2.334-19.560)	<0.0001
C-T-C	0.151	0.118	0.885 (0.657-1.192)	0.422
rs4759314A>G/rs920778 T>C				
A-T	0.767	0.685	1.000 (reference)	
A-C	0.189	0.212	1.254 (0.976-1.610)	0.076
G-T	0.024	0.061	2.742 (1.594-4.718)	<0.0001

G-C	0.020	0.042	2.410 (1.298-4.475)	0.004
rs4759314A>G/rs1899663 G>T				
A-G	0.725	0.694	1.000 (reference)	
A-T	0.231	0.203	0.920 (0.722-1.173)	0.500
G-G	0.042	0.071	1.769 (1.129-2.770)	0.012
G-T	0.003	0.032	12.910 (3.048-54.660)	<0.0001
rs4759314A>G/rs7958904 G>C				
A-G	0.723	0.686	1.000 (reference)	
A-C	0.233	0.211	0.957 (0.752-1.218)	0.719
G-G	0.013	0.069	5.510 (2.780-10.920)	<0.0001
G-C	0.031	0.034	1.169 (0.669-2.042)	0.583
rs920778 T>C/rs1899663 G>T				
T-G	0.711	0.657	1.000 (reference)	
T-T	0.080	0.089	1.216 (0.847-1.746)	0.288
C-G	0.055	0.108	2.134 (1.449-3.144)	<0.0001
C-T	0.154	0.147	1.030 (0.777-1.366)	0.836
rs920778 T>C/rs7958904 G>C				
T-G	0.702	0.641	1.000 (reference)	
T-C	0.089	0.105	1.285 (0.913-1.809)	0.149
C-G	0.034	0.113	3.642 (2.317-5.726)	<0.0001
C-C	0.175	0.141	0.885 (0.670-1.168)	0.389
rs1899663 G>T/rs7958904 G>C				
G-G	0.725	0.691	1.000 (reference)	
G-C	0.041	0.073	1.900 (1.211-2.981)	0.005
T-G	0.011	0.063	6.364 (2.992-13.540)	<0.0001
T-C	0.223	0.173	0.811 (0.630-1.045)	0.105

AOR, odds ratio; CI, confidence interval.
[a] Fisher's exact test; [b] FDR-adjusted P value.

Table S3. Combined genotype analysis for the *HOTAIR* polymorphisms rs4759314A>G, rs920778T>C, rs1899663G>T, and rs7958904G>C in RPL patients and controls.

Genotype combination	Controls (n=383)	RPL (n=403)	AOR (95% CI)	*P**
rs4759314 / rs920778				
AA/TT	223 (58.2)	190 (47.1)	1.000 (reference)	
AA/TC	118 (30.8)	112 (27.8)	1.119 (0.810-1.546)	0.495
AA/CC	10 (2.6)	20 (5.0)	2.415 (1.100-5.302)	0.028
AG/TT	11 (2.9)	35 (8.7)	3.778 (1.865-7.652)	0.0002
AG/TC	16 (4.2)	36 (8.9)	2.690 (1.445-5.006)	0.002
AG/CC	3 (0.8)	8 (2.0)	2.989 (0.779-11.471)	0.111
GG/TT	2 (0.5)	1 (0.2)	0.600 (0.054-6.672)	0.678
GG/TC	0 (0.0)	1 (0.2)	N/A	N/A
GG/CC	0 (0.0)	0 (0.0)	N/A	N/A
rs4759314 / rs1899663				
AA/GG	199 (52.0)	191 (47.4)	1.000 (reference)	
AA/GT	133 (34.7)	117 (29.0)	0.923 (0.671-1.269)	0.622
AA/TT	19 (5.0)	14 (3.5)	0.748 (0.364-1.540)	0.431
AG/GG	23 (6.0)	43 (10.7)	1.964 (1.139-3.389)	0.015
AG/GT	6 (1.6)	28 (6.9)	4.798 (1.941-11.861)	0.0007
AG/TT	1 (0.3)	8 (2.0)	8.529 (1.056-68.916)	0.044
GG/GG	2 (0.5)	1 (0.2)	0.529 (0.048-5.885)	0.605
GG/GT	0 (0.0)	1 (0.2)	N/A	N/A
GG/TT	0 (0.0)	0 (0.0)	N/A	N/A
rs4759314 / rs7958904				
AA/GG	203 (53.0)	188 (46.7)	1.000 (reference)	
AA/GC	126 (32.9)	116 (28.8)	1.000 (0.725-1.378)	0.997

AA/CC	22 (5.7)	18 (4.5)	0.894 (0.465-1.719)	0.736
AG/GG	6 (1.6)	44 (10.9)	7.874 (3.277-18.920)	<0.0001
AG/GC	18 (4.7)	27 (6.7)	1.647 (0.877-3.091)	0.120
AG/CC	6 (1.6)	8 (2.0)	1.451 (0.494-4.263)	0.498
GG/GG	1 (0.3)	0 (0.0)	N/A	N/A
GG/GC	0 (0.0)	1 (0.2)	N/A	N/A
GG/CC	1 (0.3)	1 (0.2)	1.095 (0.068-17.629)	0.949
rs920778 / rs1899663				
TT/GG	197 (51.4)	186 (46.2)	1.000 (reference)	
TT/GT	23 (6.0)	23 (5.7)	1.055 (0.571-1.949)	0.864
TT/TT	16 (4.2)	17 (4.2)	1.107 (0.542-2.259)	0.781
TC/GG	20 (5.2)	27 (6.7)	1.495 (0.808-2.768)	0.200
TC/GT	112 (29.2)	118 (29.3)	1.122 (0.809-1.557)	0.490
TC/TT	2 (0.5)	4 (1.0)	1.985 (0.357-11.028)	0.433
CC/GG	7 (1.8)	22 (5.5)	3.387 (1.411-8.134)	0.006
CC/GT	4 (1.0)	5 (1.2)	1.170 (0.306-4.475)	0.819
CC/TT	2 (0.5)	1 (0.2)	0.634 (0.056-7.251)	0.714
rs920778 / rs7958904				
TT/GG	197 (51.4)	183 (45.4)	1.000 (reference)	
TT/GC	15 (3.9)	19 (4.7)	1.381 (0.680-2.806)	0.372
TT/CC	24 (6.3)	24 (6.0)	1.087 (0.596-1.983)	0.785
TC/GG	5 (1.3)	22 (5.5)	4.792 (1.776-12.933)	0.002
TC/GC	127 (33.2)	124 (30.8)	1.057 (0.768-1.454)	0.735
TC/CC	2 (0.5)	3 (0.7)	1.646 (0.271-9.989)	0.588
CC/GG	8 (2.1)	27 (6.7)	3.614 (1.598-8.173)	0.002
CC/GC	2 (0.5)	1 (0.2)	0.566 (0.051-6.299)	0.643

CC/CC	3 (0.8)	0 (0.0)	N/A	N/A
rs1899663 / rs7958904				
GG/GG	202 (52.7)	193 (47.9)	1.000 (reference)	
GG/GC	21 (5.5)	35 (8.7)	1.826 (1.023-3.261)	0.042
GG/CC	1 (0.3)	7 (1.7)	7.735 (0.939-63.701)	0.057
GT/GG	8 (2.1)	34 (8.4)	4.293 (1.932-9.538)	0.0003
GT/GC	123 (32.1)	106 (26.3)	0.906 (0.653-1.256)	0.552
GT/CC	8 (2.1)	6 (1.5)	0.835 (0.283-2.464)	0.744
TT/GG	21 (5.5)	35 (8.7)	N/A	N/A
TT/GC	0 (0.0)	3 (0.7)	N/A	N/A
TT/CC	20 (5.2)	14 (3.5)	0.720 (0.353-1.472)	0.368

* The odds ratio was adjusted by age. RPL, recurrent pregnancy loss; AOR, adjusted odds ratio; 95% CI, 95% confidence interval.

Table S4. Differences of various clinical parameters according to *HOTAIR* gene polymorphisms in RPL patient and control subjects.

Genotypes	Creatinine (mg/dl)	TSH (uIU/mL)	BMI (kg/m²)	E2 (pg/mL)	LH (mIU/mL)	FSH (mIU/mL)	CD56+ NK cells (%)	NLR (%)	FBS (mg/dl)	TG (mg/dl)	Prolactin (ng/mL)	PLT (10^3 /μL)	PT (sec)
	Mean ± SD	Mean ± SD	Mean ± SD	Mean ± SD	Mean ± SD	Mean ± SD	Mean ± SD	Mean ± SD	Mean ± SD	Mean ± SD	Mean ± SD	Mean ± SD	Mean ± SD
**HOTAIR rs4759314**													
AA	0.68±0.15	1.93±1.37	21.45±3.35	43.91±105.44	5.43±10.56	8.07±10.00	17.24±8.06	3.42±2.44	89.04±24.11	179.25±116.08	15.81±13.48	241.73±63.39	11.08±1.80
AG	0.71±0.13	2.00±1.19	21.82±5.26	37.33±24.97	4.88±2.42	6.84±3.28	18.91±7.35	3.05±1.49	97.39±27.31	208.68±177.74	13.94±8.99	242.12±65.38	11.21±0.92
GG	0.70±0.10	1.52±0.00	21.87±3.09	16.50±0.00	2.14±0.00	4.83±0.00	18.80±0.00	3.48±1.97	96.00±1.41	65.00±0.00	6.42±0.00	225.75±49.01	10.28±1.82
P^a	0.264	0.898	0.699	0.876	0.885	0.645	0.472	0.513	0.123	0.482b	0.529	0.880	0.526
**HOTAIR rs920778**													
TT	0.69±0.15	1.92±1.45	21.74±4.12	48.73±125.15	5.62±11.67	8.02±8.85	17.68±7.67	3.38±2.20	89.97±26.19	177.61±123.25	15.74±13.50	242.45±59.12	11.01±1.56
TC	0.68±0.14	1.99±1.20	21.36±2.73	34.71±30.29	4.94±6.70	7.66±10.27	17.17±7.80	3.36±2.61	92.05±24.54	192.23±129.02	13.81±8.14	239.36±67.97	11.18±1.97
CC	0.73±0.16	1.82±0.91	20.34±4.51	34.86±17.22	5.15±3.25	7.80±5.05	24.25±12.69	3.28±1.99	92.93±15.30	179.44±129.36	20.64±21.59	250.11±83.56	11.46±1.01
P^a	0.365	0.838	0.110	0.46	0.832	0.945	0.229	0.974	0.807	0.635	0.779b	0.488b	0.326
**HOTAIR rs1899663**													
GG	0.68±0.15	1.91±1.38	21.56±3.28	43.23±76.64	5.51±11.69	7.97±8.85	17.70±7.57	3.40±2.22	89.36±26.25	177.45±124.93	16.51±15.13	241.82±58.75	10.96±1.40
GT	0.69±0.15	1.96±1.20	21.54±4.54	44.71±126.84	5.21±6.82	7.91±10.52	17.34±8.52	3.36±2.59	90.94±19.36	185.61±121.16	13.38±6.76	242.09±72.32	11.27±2.16
TT	0.69±0.11	2.10±1.81	20.77±2.86	27.93±14.95	4.51±3.05	6.72±2.99	22.00±6.56	3.10±1.69	108.08±34.25	232.23±154.09	15.30±9.34	237.44±48.51	11.43±1.04
P^a	0.855	0.837	0.633	0.763	0.895	0.849	0.618	0.851	0.044	0.297	0.941b	0.937b	0.087
**HOTAIR rs7958904**													
GG	0.69±0.15	1.91±1.41	21.56±3.29	44.72±80.66	5.81±12.12	8.08±9.12	17.58±7.67	3.29±1.95	88.12±24.91	179.12±122.04	15.78±14.52	244.38±62.16	10.95±1.43
GC	0.68±0.14	2.03±1.22	21.54±4.57	42.90±123.60	4.94±6.64	7.83±10.35	17.51±8.41	3.41±2.77	91.84±22.69	184.76±126.06	14.62±9.57	241.43±66.06	11.25±2.12
CC	0.71±0.16	1.67±1.27	20.95±2.37	31.48±19.63	4.25±2.92	6.74±2.92	20.80±6.50	3.85±2.72	105.50±29.89	199.00±149.35	16.01±10.72	223.58±57.97	11.31±1.03
P^a	0.349	0.443	0.699	0.796	0.619	0.775	0.651	0.410	0.020	0.768	0.799	0.104	0.125

Note: TSH, thyroid stimulating hormone; BMI, Body Mass Index; E2, estradiol; LH, luteinizing hormone; FSH, follicle stimulating hormone; NLR, neutrophil lymphocyte ratio; FBS, fasting blood sugar; HDL, high density lipoprotein; TG, triglyceride.
*kruskal-wallis test

Table S4. *Cont.*

aPTT (sec)	Folate (nmol/L)	Homocysteine (μmol/L)	Total cholesterol (mg/dl)	Uric acid (mg/dL)	BUN (mg/dL)	LDL cholesterol (mg/dL)	HDL cholesterol (mg/dL)	Glucose	WBC (10^3 /μL)	RBC (10^6 /μL)	Hemoglobin (g/dL)	Hematocrit (μmol/L)
**Mean ± SD**	**Mean ± SD**	**Mean ± SD**	**Mean ± SD**	**Mean ± SD**	**Mean ± SD**	**Mean ± SD**	**Mean ± SD**	**Mean ± SD**	**Mean ± SD**	**Mean ± SD**	**Mean ± SD**	**Mean ± SD**
30.77±4.18	14.31±12.86	6.96±2.55	200.66±54.30	3.84±0.94	9.72±5.37	141.03±43.72	79.45±21.98	98.81±20.25	7.72±2.81	4.16±0.41	12.41±1.32	37.13±3.58
30.60±4.63	18.77±19.31	6.93±2.26	188.42±49.73	3.77±0.92	9.49±2.29	114.50±23.23	66.83±19.78	94.86±13.54	7.22±1.89	4.17±0.46	12.50±1.23	37.36±3.40
31.76±1.91	9.58±1.31	6.66±2.23	189.50±32.22	3.80±0.42	11.10±1.81	105.00±0.00	68.00±0.00	98.67±9.07	5.97±1.66	3.90±0.33	12.18±1.10	35.20±2.57
0.846	0.413b	0.982	0.219	0.889	0.835	0.09	0.073	0.351	0.168	0.439	0.814	0.470
30.75±4.28	14.57±11.23	6.93±2.73	200.81±52.44	3.83±0.90	9.85±5.96	139.65±44.79	77.63±20.93	98.29±19.23	7.79±2.63	4.16±0.42	12.42±1.33	37.20±3.67
30.66±4.19	15.69±17.65	7.00±2.08	196.86±54.29	3.86±0.98	9.49±3.01	137.07±35.90	78.32±24.57	98.69±20.54	7.36±2.68	4.12±0.40	12.42±1.23	37.03±3.33
31.69±3.98	16.59±19.69	6.91±2.47	185.71±63.03	3.57±1.00	9.21±2.63	123.50±72.48	84.23±11.45	94.78±12.94	7.72±3.70	4.30±0.43	12.44±1.49	37.40±3.64
0.572	0.762	0.972	0.402	0.466	0.680	0.609b	0.837	0.723	0.255	0.128	0.996	0.788
30.70±4.23	14.30±9.94	6.76±2.01	199.82±53.10	3.79±0.89	9.68±5.92	137.30±44.97	78.96±20.51	97.38±18.71	7.74±2.54	4.17±0.43	12.42±1.32	37.20±3.69
30.82±4.18	16.69±19.99	7.16±2.98	196.15±55.85	3.90±0.96	9.76±3.23	139.36±40.73	76.35±25.50	99.40±21.42	7.51±3.02	4.14±0.40	12.43±1.25	37.11±3.37
31.03±4.80	12.67±8.58	7.89±3.61	205.59±39.56	3.71±1.19	9.44±2.74	145.43±28.28	77.35±11.88	101.95±13.23	7.32±2.30	4.10±0.37	12.30±1.50	36.89±3.29
0.905	0.770b	0.117	0.687	0.429	0.961	0.878	0.782	0.403	0.573	0.542	0.906	0.874
30.63±4.11	14.64±12.22	6.69±1.90	199.92±54.33	3.82±0.91	9.48±2.99	140.10±47.87	78.81±20.96	97.44±19.09	7.77±2.75	4.18±0.42	12.46±1.33	37.27±3.66
30.95±4.40	16.08±17.90	7.31±3.18	198.25±53.93	3.84±0.96	9.97±7.45	135.47±35.78	77.73±24.45	98.99±20.52	7.41±2.73	4.13±0.40	12.39±1.31	37.06±3.48
30.69±4.34	13.37±8.19	7.38±2.75	191.33±45.91	3.79±1.00	10.11±3.14	137.44±29.25	72.59±16.92	101.29±16.55	7.78±2.15	4.03±0.40	12.19±1.07	36.75±3.07
0.716	0.655	0.079	0.702	0.953	0.539	0.929b	0.694	0.492	0.344	0.081	0.514	0.560

Note: TSH, thyroid stimulating hormone; BMI, Body Mass Index; E2, estradiol; LH, luteinizing hormone; FSH, follicle stimulating hormone; NLR, neutrophil lymphocyte ratio; FBS, fasting blood sugar; HDL, high density lipoprotein; TG, triglyceride.

*kruskal-wallis test

Table S5. Differences of various clinical parameters according to *HOTAIR* gene polymorphisms in RPL patient.

Genotypes	Creatinine (mg/dl)	TSH (uIU/mL)	BMI (kg/m²)	E2 (pg/mL)	LH (mIU/mL)	FSH (mIU/mL)	CD56+ NK cells (%)	NLR	FBS (mg/dl)	TG (mg/dl)	Prolactin (ng/mL)	PLT (10^3 /μL)	PT (sec)
	**Mean ± SD**	**Mean ± SD**	**Mean ± SD**	**Mean ± SD**	**Mean ± SD**	**Mean ± SD**	**Mean ± SD**	**Mean ± SD**	**Mean ± SD**	**Mean ± SD**	**Mean ± SD**	**Mean ± SD**	**Mean ± SD**
**HOTAIR rs4759314**													
AA	0.70±0.14	1.52±0.00	20.81±1.22	16.50±0.00	2.14±0.00	4.83±0.00	17.24±8.06	2.92±1.79	96.00±1.41	65.00±0.00	6.42±0.00	219.00±83.44	11.63±0.32
AG	0.72±0.11	2.06±1.17	21.98±5.58	39.19±26.17	5.18±2.35	6.18±2.29	18.91±7.35	2.78±1.23	99.37±23.96	224.57±187.15	13.94±8.99	250.20±63.89	11.39±0.96
GG	0.73±0.13	2.17±1.50	21.37±3.35	56.42±135.61	6.63±13.32	8.14±12.77	18.80±0.00	2.95±2.18	93.80±14.04	175.79±133.71	15.81±13.48	243.74±62.58	11.33±1.79
P^a	0.793	0.828	0.468	0.680	0.731	0.589	0.472	0.878	0.142	0.254	0.529	0.667	0.943
**HOTAIR rs920778**													
TT	0.73±0.12	2.13±1.56	21.83±4.46	60.96±153.78	6.42±14.29	7.83±10.78	17.68±7.67	2.89±2.13	95.09±16.50	173.10±140.72	15.74±13.50	245.89±58.33	11.31±1.68
TC	0.73±0.12	2.23±1.29	21.20±2.52	40.73±36.07	6.29±8.30	7.57±13.18	17.17±7.80	3.02±1.96	95.48±17.78	217.83±160.27	13.81±8.14	240.90±63.25	11.37±1.70
CC	0.75±0.11	1.80±0.83	20.23±4.88	36.76±18.36	5.48±3.36	7.63±5.39	24.25±12.69	2.70±1.60	92.93±15.30	126.80±77.16	20.64±21.59	258.12±96.68	11.58±0.80
P^a	0.752	0.565	0.082	0.497	0.961	0.988	0.229	0.805	0.868	0.213	0.779b	0.617b	0.84
**HOTAIR rs1899663**													
GG	0.73±0.13	2.11±1.46	21.54±3.29	50.77±90.95	6.16±13.89	7.80±10.49	17.70±7.57	2.96±2.17	94.78±15.88	179.23±145.39	16.51±15.13	247.49±61.64	11.32±1.67
GT	0.74±0.12	2.16±1.32	21.50±4.80	58.14±165.60	6.62±8.50	7.98±13.61	17.34±8.52	2.96±1.88	93.36±13.09	196.53±146.81	13.38±6.76	242.13±66.40	11.37±1.74
TT	0.72±0.11	2.49±1.91	20.71±3.02	34.00±16.15	6.01±3.61	4.87±2.25	22.00±6.56	2.19±1.17	108.08±34.25	181.00±166.77	15.30±9.34	233.69±52.37	11.50±0.69
P^a	0.792	0.644	0.683	0.807	0.962	0.719	0.618	0.438	0.496b	0.846	0.941b	0.611	0.9
**HOTAIR rs7958904**													
GG	0.73±0.12	2.12±1.51	21.49±3.29	53.18±95.98	6.51±14.40	7.98±10.83	17.58±7.67	2.70±1.39	93.25±14.53	186.43±147.79	15.78±14.52	249.25±64.76	11.29±1.71
GC	0.73±0.12	2.27±1.31	21.56±4.83	54.23±159.63	6.28±8.24	7.81±13.29	17.51±8.41	3.21±2.48	95.27±15.05	181.89±140.28	14.62±9.57	244.25±57.71	11.38±1.72
CC	0.72±0.14	1.80±1.35	20.98±2.55	38.77±22.53	4.92±3.59	5.25±2.61	20.80±6.50	3.27±3.38	105.50±29.89	176.20±164.31	16.01±10.72	214.33±66.37	11.53±0.81
P^a	0.807	0.421	0.808	0.893	0.889	0.683	0.651	0.741b	0.278b	0.973	0.799	0.056	0.791

Note: TSH, thyroid stimulating hormone; BMI, Body Mass Index; E2, estradiol; LH, luteinizing hormone; FSH, follicle stimulating hormone; NLR, neutrophil lymphocyte ratio; FBS, fasting blood sugar; HDL, high density lipoprotein; TG, triglyceride.
*kruskal-wallis test

Table S5. *Cont.*

aPTT (sec)	Folate (nmol/L)	Homocysteine (μmol/L)	Total cholesterol (mg/dl)	Uric acid (mg/dL)	BUN (mg/dL)	LDL cholesterol (mg/dL)	HDL cholesterol (mg/dL)	Glucose (mg/dL)	WBC (10^3 /μL)	RBC (10^6 /μL)	Hemoglobin (g/dL)	Hematocrit (μmol/L)
Mean ± SD	Mean ± SD	Mean ± SD	Mean ± SD	Mean ± SD	Mean ± SD	Mean ± SD	Mean ± SD	Mean ± SD	Mean ± SD	Mean ± SD	Mean ± SD	Mean ± SD
33.18±0.53	9.58±1.31	6.66±2.23	162.00±4.24	3.80±0.42	11.00±2.55	105.00±0.00	68.00±0.00	95.00±0.00	4.65±0.52	3.94±0.11	12.70±0.57	36.20±0.42
30.72±4.79	19.06±19.40	6.90±2.26	192.83±51.46	3.79±0.95	9.41±2.18	106.50±25.32	61.20±13.74	94.16±9.32	7.25±1.99	4.19±0.48	12.57±1.30	37.56±3.49
32.23±4.23	14.30±13.30	6.91±2.02	186.01±45.81	3.84±0.85	10.60±6.90	110.84±39.46	59.32±16.74	97.17±14.35	7.37±3.00	4.23±0.41	12.61±1.35	37.59±3.83
0.065	0.289b	0.985	0.500	0.937	0.469	0.961	0.84	0.424	0.394	0.563	0.970	0.873
31.91±4.52	14.82±11.77	6.89±2.09	190.34±43.37	3.78±0.82	10.78±7.80	109.65±36.79	59.15±15.08	97.32±15.14	7.31±2.57	4.25±0.41	12.64±1.35	37.71±3.80
31.90±4.18	15.49±17.89	6.93±1.96	185.55±53.19	3.97±0.95	9.79±2.75	125.50±25.00	56.71±13.87	95.59±10.46	7.32±3.04	4.15±0.43	12.57±1.26	37.38±3.67
32.79±4.01	17.24±20.33	6.91±2.54	168.81±36.03	3.45±0.70	9.73±2.45	77.50±26.16	87.55±16.19	95.09±13.94	7.47±4.01	4.37±0.44	12.49±1.65	37.38±3.79
0.774	0.830	0.991	0.204	0.084	0.456	0.305	0.033	0.616	0.978	0.078	0.867	0.768
31.84±4.48	14.69±10.42	6.85±1.95	189.54±46.48	3.75±0.83	10.53±7.74	101.71±31.86	61.29±16.97	96.41±14.12	7.43±2.83	4.22±0.45	12.53±1.41	37.48±3.97
32.04±4.07	16.59±20.53	6.86±1.92	181.19±47.91	3.97±0.93	10.10±2.78	138.00±33.94	52.93±8.90	96.29±12.47	7.24±2.95	4.23±0.37	12.76±1.15	37.87±3.32
32.50±5.14	10.44±4.53	7.99±3.72	203.91±39.39	3.72±0.69	10.29±2.11	146.00±38.74	75.45±14.35	100.08±13.64	6.73±2.00	4.06±0.43	12.45±1.60	36.78±3.85
0.826	0.370	0.893b	0.206	0.180	0.879	0.057	0.128	0.633	0.640	0.363	0.377	0.485
31.83±4.28	15.10±12.96	6.76±1.82	187.76±43.54	3.79±0.85	10.07±2.95	101.59±34.80	60.93±16.55	96.30±14.67	7.23±2.75	4.25±0.44	12.61±1.39	37.61±3.91
32.11±4.40	15.91±18.17	7.05±2.28	184.92±52.48	3.92±0.93	10.77±9.81	116.00±30.33	55.25±14.02	96.47±11.21	7.46±3.08	4.21±0.38	12.64±1.32	37.65±3.63
31.97±4.87	11.93±6.00	7.44±2.82	193.11±44.88	3.74±0.65	10.78±2.72	135.75±37.70	66.45±15.84	99.24±15.93	7.33±2.10	4.02±0.44	12.40±0.84	36.94±3.06
0.878	0.624	0.283	0.772	0.506	0.67	0.205	0.205	0.702	0.834	0.105	0.788	0.721

Note: TSH, thyroid stimulating hormone; BMI, Body Mass Index; E2, estradiol; LH, luteinizing hormone; FSH, follicle stimulating hormone; NLR, neutrophil lymphocyte ratio; FBS, fasting blood sugar; HDL, high density lipoprotein; TG, triglyceride.
*kruskal-wallis test

Table S6. Differences of various clinical parameters according to *HOTAIR* gene polymorphisms in control

**Genotypes**	**Creatinine (mg/dl)**	**TSH (uIU/mL)**	**BMI (kg/m^2)**	**E2 (pg/mL)**	**LH (mIU/mL)**	**FSH (mIU/mL)**	**PLT (10^3 /µL)**	**PT (sec)**	**aPTT (sec)**	**Folate (nmol/L)**
	**Mean ± SD**	**Mean ± SD**	**Mean ± SD**	**Mean ± SD**	**Mean ± SD**	**Mean ± SD**	**Mean ± SD**	**Mean ± SD**	**Mean ± SD**	**Mean ± SD**
**HOTAIR rs4759314**										
AA	0.63±0.15	1.54±1.02	21.69±3.33	26.00±15.00	3.62±2.57	7.98±2.86	240.30±64.02	10.81±1.77	29.25±3.56	14.38±9.73
AG	0.68±0.18	1.66±1.34	20.84±2.43	25.93±11.12	3.29±2.24	9.98±5.24	224.04±66.34	10.68±0.56	30.24±4.25	4.81±0.00
GG	0.70±0.00	0.00±0.00	22.94±4.76	0.00±0.00	0.00±0.00	0.00±0.00	232.50±7.78	8.93±1.59	30.35±1.63	0.00±0.00
P^a	0.324	0.755	0.591	0.990	0.726	0.062	0.470	0.292	0.480	0.342
**HOTAIR rs920778**										
TT	0.64±0.16	1.54±1.12	21.51±3.17	26.07±14.48	4.15±3.09	8.35±3.45	239.79±59.72	10.69±1.36	29.54±3.65	12.93±6.82
TC	0.62±0.14	1.56±0.88	22.24±3.62	25.92±15.53	2.90±1.38	7.79±2.63	238.08±71.88	10.91±2.28	28.86±3.53	17.94±15.47
CC	0.68±0.23	1.98±1.88	20.76±3.06	25.35±3.32	2.90±0.71	8.90±2.26	236.50±56.83	11.28±1.33	29.90±3.44	8.19±0.00
P^a	0.473	0.585	0.470	0.997	0.029	0.585	0.965	0.454	0.365	0.654b
**HOTAIR rs1899663**										
GG	0.63±0.16	1.56±1.16	21.63±3.28	26.57±14.63	4.09±3.29	8.32±3.48	236.97±55.87	10.54±0.81	29.40±3.52	11.57±4.82
GT	0.64±0.16	1.61±0.84	21.68±3.37	26.36±15.64	3.15±1.46	7.81±2.75	242.05±76.78	11.15±2.59	29.33±3.85	17.56±14.53
TT	0.64±0.11	0.81±0.20	21.08±2.33	21.25±10.59	3.02±1.30	8.57±2.48	240.78±46.08	11.29±1.56	28.29±2.55	35.05±0.00
P^a	0.972	0.347	0.942	0.569	0.124	0.616	0.946b	0.024	0.697	0.242b
**HOTAIR rs7958904**										
GG	0.64±0.16	1.53±1.12	21.78±3.30	26.43±14.78	4.27±3.33	8.29±3.56	240.19±59.70	10.56±0.87	29.30±3.48	11.71±4.77
GC	0.62±0.13	1.62±0.94	21.46±3.43	26.49±15.80	2.92±1.36	7.86±2.77	239.24±72.02	11.09±2.54	29.50±3.97	17.94±15.47
CC	0.71±0.20	1.29±1.01	20.85±1.85	22.50±10.30	3.47±1.73	8.45±2.28	230.78±50.62	10.95±1.25	28.69±2.29	35.05±0.00
P^a	0.131	0.747	0.729	0.665	0.024	0.709	0.776	0.077	0.722	0.261b

Note: TSH, thyroid stimulating hormone; BMI, Body Mass Index; E2, estradiol; LH, luteinizing hormone; FSH, follicle stimulating hormone.
*kruskal-wallis test

Table S6. *Cont.*

**Homocysteine (μmol/L)** Mean ± SD	**Total cholesterol (mg/dl)** Mean ± SD	**Uric acid (mg/dL)** Mean ± SD	**BUN (mg/dL)** Mean ± SD	**LDL cholesterol (mg/dL)** Mean ± SD	**HDL cholesterol (mg/dL)** Mean ± SD	**Glucose** Mean ± SD	**WBC (10^3 /μL)** Mean ± SD	**RBC (10^6 /μL)** Mean ± SD	**Hemoglobin (g/dL)** Mean ± SD	**Hematocrit (μmol/L)** Mean ± SD
7.38±5.29	215.55±58.20	3.83±1.04	8.87±3.07	146.76±42.27	85.13±19.88	100.31±24.37	8.07±2.58	4.09±0.39	12.21±1.26	36.81±3.36
7.69±2.71	176.67±43.97	3.72±0.83	9.72±2.64	122.50±19.81	73.16±24.34	96.75±21.46	7.16±1.63	4.11±0.40	12.31±1.04	36.92±3.20
0.00±0.00	217.00±8.49	0.00±0.00	11.30±0.00	0.00±0.00	0.00±0.00	100.50±12.02	7.29±1.00	3.85±0.55	11.65±1.48	34.20±3.96
0.937	0.023	0.701	0.387	0.167	0.108	0.851	0.295	0.677	0.767	0.539
7.20±5.69	211.59±58.61	3.89±1.00	8.91±2.93	148.74±43.21	85.07±18.22	99.20±22.46	8.25±2.61	4.08±0.41	12.20±1.28	36.81±3.53
8.09±3.58	211.21±52.62	3.71±1.02	9.10±3.29	138.29±36.90	83.23±23.89	102.33±27.75	7.42±2.16	4.08±0.37	12.22±1.18	36.74±3.01
7.00±0.00	239.80±100.98	3.90±1.60	8.23±2.84	169.50±81.32	80.90±9.33	94.29±12.24	8.23±3.16	4.16±0.40	12.33±1.19	37.43±3.56
0.926	0.558	0.597	0.723	0.336	0.609b	0.549	0.062	0.877	0.960	0.819
6.01±2.43	211.14±57.65	3.84±0.98	8.78±2.64	148.80±42.65	86.18±17.24	98.38±22.49	8.06±2.16	4.12±0.39	12.30±1.22	36.96±3.41
11.17±8.36	214.27±59.63	3.82±1.00	9.36±3.64	139.43±41.46	81.79±25.05	102.51±27.36	7.82±3.10	4.02±0.40	12.06±1.26	36.54±3.32
6.40±0.00	208.67±43.45	3.68±1.88	7.96±3.21	145.00±24.45	78.30±12.77	105.00±12.81	8.24±2.54	4.17±0.26	12.08±1.38	36.99±2.80
0.150b	0.926	0.642b	0.255	0.555	0.450	0.415	0.754	0.173	0.342	0.524
6.13±2.47	213.71±61.77	3.87±1.00	8.84±2.92	150.84±45.61	85.86±18.20	98.58±22.65	8.34±2.64	4.12±0.39	12.30±1.23	36.98±3.42
10.87±8.53	213.17±51.89	3.75±0.99	9.09±3.17	137.90±35.99	83.10±23.39	101.74±27.09	7.36±2.27	4.04±0.41	12.11±1.25	36.60±3.29
6.40±0.00	188.27±49.71	3.90±1.56	9.16±3.53	138.80±25.31	76.68±17.74	103.79±17.53	8.32±2.15	4.04±0.35	11.95±1.28	36.60±3.13
0.409b	0.372	0.752	0.821	0.295	0.503	0.552	0.014	0.295	0.361	0.577

Note: TSH, thyroid stimulating hormone; BMI, Body Mass Index; E2, estradiol; LH, luteinizing hormone; FSH, follicle stimulating hormone.
*kruskal-wallis test

Table S7. Differences of various clinical parameters according to *HOTAIR* gene polymorphisms in RPL patient.

Genotypes	CD3	CD4	CD8	CD19	PAI-1
	**Mean ± SD**	**Mean ± SD**	**Mean ± SD**	**Mean ± SD**	**Mean ± SD**
***HOTAIR* rs4759314**					
AA	67.56±8.04	36.74±7.21	27.99±7.58	12.51±4.50	11.53±8.65
AG	66.31±11.04	35.31±7.87	27.86±7.97	12.91±5.82	10.83±5.73
GG	75.20±0.00	40.30±0.00	31.70±0.00	5.80±0.00	10.16±5.36
P^a	0.559	0.613	0.886	0.359	0.881
***HOTAIR* rs920778**					
TT	66.79±9.68	35.65±8.09	28.75±8.87	12.82±5.19	11.48±6.79
TC	68.20±7.64	37.52±6.47	27.26±5.68	12.53±4.38	11.19±9.37
CC	64.33±11.06	34.33±2.89	25.00±8.54	7.67±4.04	10.41±5.97
P^a	0.623	0.405	0.499	0.204	0.928
***HOTAIR* rs1899663**					
GG	68.34±6.92	35.88±7.14	29.39±8.35	12.95±4.95	10.94±6.04
GT	66.26±10.65	37.49±7.52	25.90±5.83	12.21±4.66	11.97±10.22
TT	57.00±19.80	32.00±11.31	27.00±9.90	6.50±2.12	9.73±2.65
P^a	0.130	0.395	0.081	0.159	0.728
***HOTAIR* rs7958904**					
GG	68.59±7.14	36.55±7.88	28.91±8.66	12.79±5.04	10.75±5.78
GC	67.14±7.82	36.92±5.87	27.21±5.47	12.72±4.39	12.04±10.38
CC	46.33±23.18	27.00±11.79	22.00±11.14	5.33±2.52	11.87±5.91
P^a	0.192*	0.075	0.425*	0.032	0.660

Note: CD3, cluster of differentiation 3; PAI-1, plasminogen activator inhibitor-1.

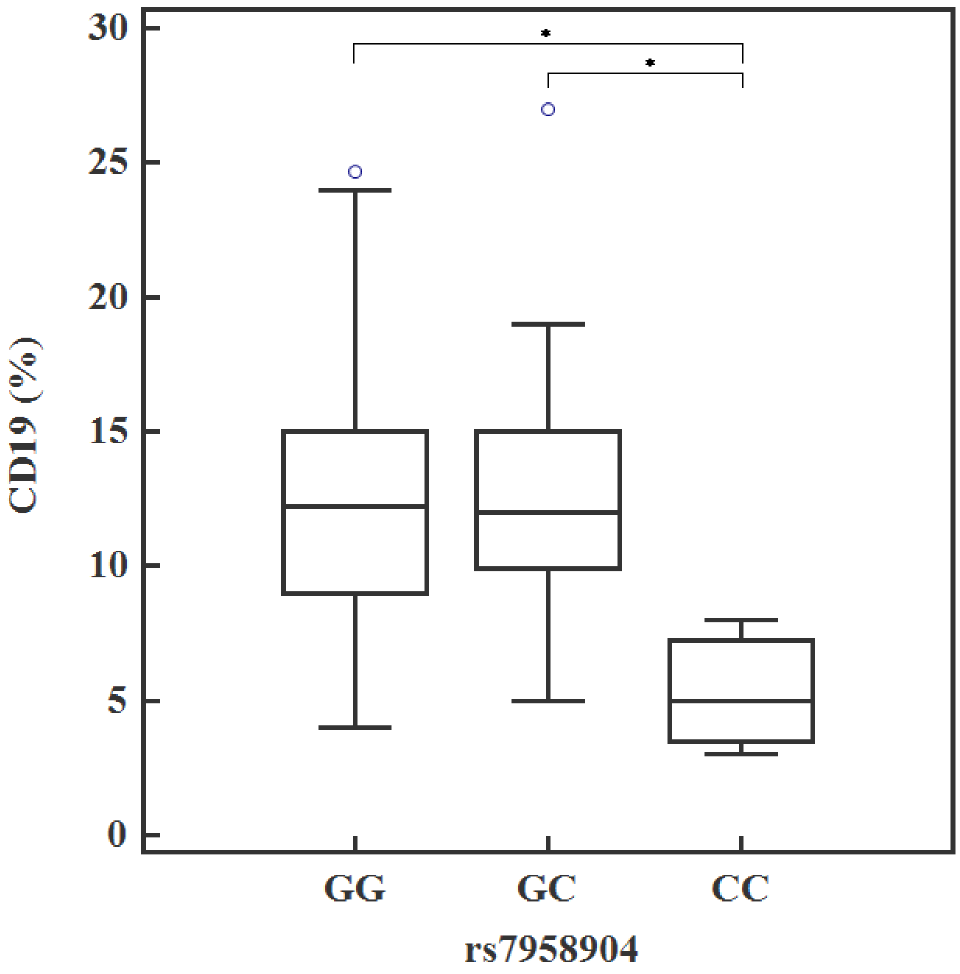

Supplementary Figure S1. Association between differences in CD19 proportions, and the *HOTAIR* rs7958904G>C polymorphisms in patients with recurrent pregnancy loss. * $p < 0.05$.
